# The Role of Immunologic Factors in Endometrial Receptivity: An Embryo–Endometrium Dialogue

**DOI:** 10.3390/ijms27104588

**Published:** 2026-05-20

**Authors:** Evangelia Panagodimou, Ianthi Terzopoulou, Olga Triantafyllidou, Georgios Markantes, Neoklis Georgopoulos, Nikolaos Vlahos, George Adonakis, Apostolos Kaponis

**Affiliations:** 1 Department of Obstetrics & Gynecology, IASO Clinic, 151 23 Athens, Greece; evakipanagod@gmail.com; 2 Department of Obstetrics & Gynecology, Medical School, University of Patras, 262 21 Patron, Greece; ianthi.ch.t@gmail.com (I.T.); gmarkantes@hotmail.com (G.M.); neoklisgeorgo@gmail.com (N.G.); adonakis@upatras.gr (G.A.); 3 Department of Obstetrics & Gynecology, Aretaieio Hospital, Medical School of Athens, 115 28 Athens, Greece; triantafyllidouolga@gmail.com (O.T.); nikosvlachos@med.ua.gr (N.V.)

**Keywords:** endometrial receptivity, embryo implantation, uterine natural killer cells, regulatory T cells, embryo–endometrium communication, reproductive immunology, cytokines, signaling pathways, recurrent implantation failure

## Abstract

Successful embryo implantation requires dynamic, bidirectional communication between a developmentally competent blastocyst and a receptive endometrium, integrating hormonal, molecular, and immunologic signals. Increasing evidence indicates that endometrial receptivity is critically dependent on a specialized immune microenvironment that supports trophoblast invasion while maintaining maternal tolerance. This review synthesizes current knowledge on the immunologic regulation of implantation, with emphasis on uterine natural killer (uNK) cells, regulatory T cells (Tregs), macrophages, dendritic cells, and cytokine networks. We further examine intracellular signaling pathways—including JAK/STAT, PI3K/AKT, NF-κB, and MAPK—that integrate immune and decidual responses. The bidirectional embryo–endometrium dialogue is explored through embryo-derived mediators such as human chorionic gonadotropin (hCG), cytokines, growth factors, and extracellular vesicles. The endometrium is increasingly recognized as a biosensor of embryo quality, selectively supporting viable embryos. Disruption of this complex communication network is implicated in recurrent implantation failure and early pregnancy loss. Despite substantial mechanistic advances, clinical translation remains limited. Emerging strategies, including immune profiling, microbiome modulation, and extracellular vesicle-based diagnostics, hold promise for precision reproductive medicine.

## 1. Introduction

Embryo implantation is a highly coordinated biological process requiring synchronization between a developmentally competent blastocyst and a receptive endometrium [1,2,3]. Despite significant advances in assisted reproductive technologies (ARTs), implantation failure remains a major limiting factor in achieving successful pregnancy outcomes [4]. While early research emphasized hormonal and structural determinants of implantation, it is now clear that immune regulation plays a central and indispensable role in establishing endometrial receptivity [5,6,7].

During the mid-luteal phase, the endometrium undergoes progesterone-driven decidualization, characterized by stromal cell differentiation, glandular secretion, and expression of adhesion molecules such as integrins [8,9]. These changes define the “window of implantation,” a temporally restricted period during which the endometrium becomes receptive to embryo attachment. Beyond structural readiness, this phase is marked by a finely regulated immune environment that enables tolerance of the semi-allogeneic embryo while maintaining protection against pathogens [10].

This review examines the immunologic mechanisms underlying endometrial receptivity, integrating cellular, molecular, and signaling perspectives. Particular emphasis is placed on immune cell populations, cytokine networks, intracellular pathways, and the bidirectional communication between embryo and endometrium. The clinical implications of these processes for implantation failure and emerging therapeutic strategies are also discussed.

## 2. Literature Search Strategy

A comprehensive literature search was conducted using PubMed, Scopus, and Web of Science databases. Search terms included “endometrial receptivity”, “implantation immunology”, “uterine natural killer cells”, “regulatory T cells”, “embryo–endometrium communication”, “recurrent implantation failure”, “decidualization”, “extracellular vesicles and implantation”, “endometrial microbiome”, and “cytokine signaling in pregnancy”. Articles published between 2000 and 2025 were prioritized, with particular emphasis on mechanistic, translational, and randomized clinical studies. Seminal foundational studies published prior to 2000 were included where they provided essential conceptual context. Both human and animal (primarily murine) studies were considered. Review articles, original research, and meta-analyses were included. Studies were evaluated for methodological quality, relevance to human reproductive biology, and recency of findings. Where evidence was conflicting, the most rigorously designed studies were preferentially cited.

## 3. Immunologic Landscape of the Receptive Endometrium

### 3.1. Uterine Natural Killer Cells

Uterine natural killer (uNK) cells represent the dominant leukocyte population in the receptive endometrium, accounting for up to 40% of stromal cells during the window of implantation [11,12]. In contrast to peripheral NK cells, which exhibit strong cytotoxic activity, uNK cells display a CD56^bright^ CD16^−^ phenotype and function primarily as immunoregulatory and pro-angiogenic cells [13].

uNK cells play a critical role in regulating trophoblast invasion and spiral artery remodeling through secretion of cytokines, chemokines, and angiogenic factors such as vascular endothelial growth factor (VEGF) and placental growth factor (PlGF) [14,15,16]. Their activity is modulated through interactions between maternal killer-cell immunoglobulin-like receptors (KIRs) and fetal HLA-C molecules expressed by extravillous trophoblasts [17,18]. Specific KIR–HLA combinations influence implantation success and placental development, with certain genotypes associated with adverse pregnancy outcomes [19].

Importantly, functional dysregulation of uNK cells—rather than absolute cell number—appears to be the key determinant of implantation failure. Altered cytokine secretion profiles, impaired angiogenic signaling, and defective communication with decidual stromal cells may all contribute to reproductive pathology [20]. Clinical assessment of uNK cells typically involves endometrial biopsy and immunohistochemical quantification; however, the predictive value of such assessments remains a subject of debate, as reference ranges have not been standardized across laboratories and populations. Furthermore, elevated uNK cell counts in isolation do not reliably predict outcomes, reinforcing the importance of functional characterization over enumeration alone.

### 3.2. Regulatory T Cells

Regulatory T cells (Tregs), characterized by expression of FOXP3, are essential for maintaining maternal–fetal immune tolerance [21]. They suppress effector T-cell responses and regulate antigen-presenting cells, thereby preventing immune-mediated rejection of the embryo.

Treg expansion during early pregnancy is influenced by progesterone, local cytokines, and exposure to paternal antigens [22]. Seminal factor exposure during conception, including through unprotected intercourse prior to IVF, has been proposed to prime Treg-mediated tolerance, potentially explaining the epidemiological association between longer cohabitation and improved IVF outcomes. Reduced Treg number or function has been associated with recurrent implantation failure and recurrent pregnancy loss [23]. Mechanistically, Tregs contribute to implantation by secreting immunosuppressive cytokines such as IL-10 and TGF-β, suppressing effector CD4+ and CD8+ T-cell responses, and modulating dendritic cell activation at the maternal–embryonic interface. Emerging evidence also suggests cross-talk between Tregs and uNK cells, whereby Treg-derived IL-10 may enhance the pro-angiogenic function of uNK cells, further integrating the immunologic circuitry of the receptive endometrium [24].

### 3.3. Macrophages and Dendritic Cells

Macrophages and dendritic cells are key regulators of tissue remodeling, angiogenesis, and immune tolerance at the maternal–fetal interface. Decidual macrophages, which constitute approximately 20–30% of decidual leukocytes, typically exhibit an M2-like anti-inflammatory phenotype during the implantation window [25]. These cells produce IL-10, insulin-like growth factor-1 (IGF-1), and various matrix metalloproteinases (MMPs) that contribute to extracellular matrix remodeling, trophoblast invasion, and spiral artery adaptation. The M1/M2 polarization of decidual macrophages is dynamically regulated by local cytokines, progesterone, and embryo-derived signals, with a shift toward M1 pro-inflammatory activity implicated in implantation failure and early pregnancy loss.

Dendritic cells (DCs) play a complementary role at the maternal–fetal interface by modulating antigen presentation, T-cell priming, and the induction of peripheral tolerance. Uterine DCs are particularly enriched in the peri-implantation endometrium and undergo progesterone-driven tolerogenic maturation, acquiring the capacity to induce Treg differentiation and suppress pro-inflammatory effector responses [26]. Plasmacytoid DCs additionally contribute innate immune sensing functions, responding to embryonic danger signals while maintaining immune homeostasis. Dysregulation of decidual DC populations—through impaired tolerogenic maturation or excessive activation—may compromise the immunologic balance required for successful implantation, contributing to the pro-inflammatory milieu observed in recurrent implantation failure [27].

### 3.4. Cytokine Networks

Implantation occurs within a tightly controlled and temporally regulated inflammatory environment. Contrary to the earlier notion that inflammation is inherently detrimental to implantation, it is now understood that a transient, controlled pro-inflammatory state during the attachment phase is necessary to facilitate blastocyst adhesion, trophoblast invasion, and the initiation of decidualization [28]. This is followed by a progressive shift toward anti-inflammatory and tolerogenic signaling to sustain placental development and maternal immune tolerance. Perturbation of this sequential inflammatory program—through excessive or prolonged pro-inflammatory signaling, or conversely, premature immunosuppression—is associated with implantation failure and adverse pregnancy outcomes [29].

Cytokines such as leukemia inhibitory factor (LIF), interleukin-10 (IL-10), and transforming growth factor-β (TGF-β) are critical regulators of decidualization and immune balance [30]. The classical Th1/Th2 paradigm is now recognized as overly simplistic, with additional roles for Th17 cells and regulatory pathways contributing to the dynamic immune landscape [31].

## 4. Molecular and Intracellular Signaling Pathways

The integration of immune and hormonal signals in the endometrium is mediated through a network of intracellular signaling pathways that regulate gene expression, cellular differentiation, metabolic adaptation, and immune responses. These pathways do not operate in isolation; rather, they form an interconnected signaling web in which cytokine receptors, steroid hormone receptors, and growth factor receptors converge to coordinate the endometrial response to implantation cues. Understanding these molecular mechanisms is critical not only for elucidating the biology of implantation but also for identifying potential therapeutic targets in women with recurrent implantation failure.

The Janus kinase/signal transducer and activator of transcription (JAK/STAT) pathway is a central transducer of cytokine signaling in the endometrium, particularly in response to leukemia inhibitory factor (LIF), IL-6, and oncostatin M [32]. LIF signaling through STAT3 is indispensable for uterine receptivity in rodent models, and disruption of this pathway abolishes implantation. In human endometrium, STAT3 phosphorylation is maximal during the window of implantation and is required for the expression of implantation-associated genes including glycodelin and mucin-1 [33]. The JAK/STAT pathway additionally mediates uNK cell activation and Treg differentiation, linking cytokine and immune regulation at the molecular level. The phosphoinositide 3-kinase (PI3K)/AKT pathway regulates stromal cell survival, proliferation, and metabolic adaptation during decidualization [34,35]. AKT activation downstream of insulin, IGF-1, and EGF receptors promotes glucose uptake, protein synthesis, and the anti-apoptotic gene expression profile characteristic of decidualized stromal cells. Dysregulation of PI3K/AKT signaling has been implicated in endometrial insulin resistance, a condition increasingly recognized in women with polycystic ovary syndrome and recurrent implantation failure.

Nuclear factor kappa B (NF-κB) is a key regulator of inflammatory responses, controlling the expression of cytokines and adhesion molecules involved in implantation [36]. Mitogen-activated protein kinase (MAPK) pathways contribute to cellular proliferation, differentiation, and stress responses. Dysregulation of these signaling networks may disrupt the coordination of immune and endocrine signals, contributing to implantation failure [37,38].

## 5. Embryo–Endometrium Communication

### 5.1. Embryo-Derived Signals

Implantation is fundamentally a bidirectional process in which the embryo actively communicates with and modifies the endometrial environment, rather than passively responding to a permissive uterine state. Preimplantation embryos secrete a diverse repertoire of bioactive molecules beginning as early as the zygote stage, with secretory activity increasing substantially as development proceeds through cleavage stages to the blastocyst [39]. These embryo-derived signals serve multiple functions: they prepare the endometrium for receptivity, modulate local immune responses, promote vascular remodeling, and facilitate the adhesion and invasion processes essential for successful implantation. The nature and quantity of these signals reflect the developmental competence of the embryo, providing a molecular basis for the endometrium’s capacity to discriminate between embryos of differing quality.

Human chorionic gonadotropin (hCG) is one of the earliest and most well-characterized embryo-derived signals, detectable in embryo culture media from the late cleavage stage onward [40]. Beyond its classical role in sustaining the corpus luteum and progesterone production, hCG exerts direct effects on the endometrium by promoting decidualization of stromal cells, enhancing angiogenesis through upregulation of VEGF, and modulating decidual immune cell function toward a tolerogenic phenotype. hCG engages luteinizing hormone/chorionic gonadotropin receptors (LHCGRs) expressed on endometrial stromal cells, uNK cells, and macrophages, and has been shown to promote Treg expansion and reduce NK cell cytotoxicity, thereby fostering the immunologic conditions necessary for tolerance. Notably, low-dose intrauterine hCG instillation prior to embryo transfer has been explored as a therapeutic strategy to enhance endometrial receptivity, though results from clinical trials have been mixed [40].

Interleukin-1β (IL-1β) is another pivotal embryo-derived mediator, secreted by the blastocyst in quantities that correlate with embryo developmental potential [41]. IL-1β acts on endometrial epithelial cells to upregulate integrin αvβ3, ICAM-1, and other adhesion molecules required for blastocyst attachment. It also activates NF-κB in endometrial stromal cells, promoting the production of LIF, IL-6, and colony-stimulating factor-1 (CSF-1), which collectively support trophoblast invasion and immune modulation. Heparin-binding epidermal growth factor-like growth factor (HB-EGF) represents a further critical embryonic signal that acts in a paracrine manner to stimulate endometrial ErbB receptor signaling, promoting epithelial remodeling, mucin shedding, and pinopode retraction to expose the adhesive epithelial surface [30]. Together, these embryo-derived mediators orchestrate a coordinated molecular response in the endometrium that converts a structurally prepared but non-adhesive surface into one capable of supporting blastocyst attachment and trophoblast invasion.

### 5.2. Extracellular Vesicles

Extracellular vesicles (EVs), encompassing exosomes (30–150 nm), microvesicles (100–1000 nm), and apoptotic bodies, have emerged as indispensable mediators of the molecular dialogue between the embryo and the endometrium [42,43]. EVs carry a complex cargo of microRNAs (miRNAs), messenger RNAs (mRNAs), proteins, lipids, and metabolites that are selectively packaged to reflect the physiological state of the originating cell. Upon uptake by recipient cells through membrane fusion, endocytosis, or receptor-mediated interactions, EV cargo is delivered intracellularly, where it can directly regulate gene expression, signal transduction, and immune function. This mode of intercellular communication is particularly suited to the implantation microenvironment, where epithelial, stromal, immune, and embryonic cells must coordinate over short distances and across fluid-filled spaces [44].

Embryo-derived EVs have been identified in conditioned media from preimplantation embryos cultured in vitro, and their miRNA cargo has been shown to modulate endometrial epithelial gene expression, enhancing receptivity markers and promoting a tolerogenic immune environment [43,45]. Specific miRNAs enriched in embryo-derived EVs, including miR-191, miR-372, and members of the let-7 family, target pathways involved in cell adhesion, cytokine production, and Wnt signaling. Conversely, endometrial-derived EVs—secreted by epithelial and stromal cells under progesterone stimulation—deliver cargo to the trophoblast that promotes adhesion, invasion, and immune evasion. This bidirectional EV exchange constitutes a dynamic molecular communication channel that operates in parallel with soluble cytokine and growth factor signaling. The composition of endometrial EVs changes dynamically across the menstrual cycle, with a distinct pro-implantation EV profile emerging during the window of implantation [46].

Dysregulation of EV biogenesis, cargo loading, or uptake mechanisms may impair endometrial receptivity and contribute to implantation failure. Altered miRNA profiles in endometrial or serum EVs have been reported in women with recurrent implantation failure, endometriosis, and polycystic ovary syndrome, suggesting their potential utility as non-invasive biomarkers of endometrial function [42]. Furthermore, the therapeutic potential of engineered or naturally derived EVs to deliver pro-receptivity signals to the endometrium represents an exciting frontier in precision reproductive medicine, though clinical translation remains at an early stage.

### 5.3. Endometrium as a Biosensor

**Figure 1:**
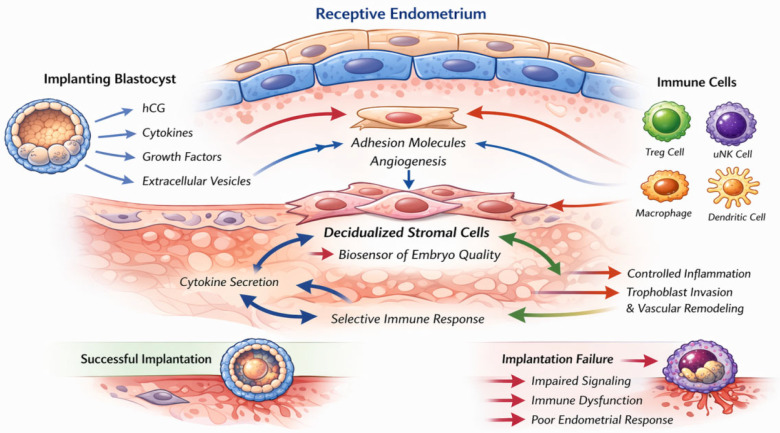
Schematic representation of bidirectional signaling between the implanting blastocyst and the receptive endometrium during the window of implantation. Embryo-derived factors, including human chorionic gonadotropin, cytokines, growth factors, and extracellular vesicles, modulate endometrial epithelial and stromal cells, promoting decidualization, adhesion molecule expression, and angiogenesis. Decidualized stromal cells function as biosensors of embryo quality, adjusting cytokine secretion and immune cell recruitment accordingly. Uterine natural killer cells, regulatory T cells, macrophages, and dendritic cells establish a state of controlled inflammation and immune tolerance that supports trophoblast invasion and vascular remodeling.

One of the most significant conceptual advances in reproductive biology over the past decade is the recognition of the endometrium as an active biosensor of embryo quality, rather than a passive scaffold awaiting implantation. This biosensor model, grounded in seminal in vitro work by Teklenburg et al. and subsequently elaborated in both animal and human studies, suggests that decidualized endometrial stromal cells possess the molecular machinery to evaluate the developmental competence of an opposing blastocyst and cause a differentiated response accordingly [47,48]. Competent embryos elicit a pro-implantation response characterized by sustained decidualization, enhanced secretion of pro-angiogenic cytokines, and Treg recruitment. By contrast, developmentally compromised embryos—including those with aneuploidy, arrested development, or metabolic deficiencies—provoke an inflammatory response leading to stromal cell apoptosis and embryo rejection (Figure 1).

The molecular basis of endometrial biosensing involves multiple pathways. Embryo-derived signals, including hCG, IL-1β, and EVs, are integrated by decidual stromal cells through pattern recognition-like mechanisms that accordingly activate pro-survival or pro-apoptotic cascades depending on signal quality and intensity. FOXO transcription factors, p53 signaling, and the unfolded protein response have all been implicated in the decidual quality-control response. Crucially, this biosensor mechanism is itself regulated by the decidualization process: only fully decidualized stromal cells acquire the discriminatory capacity to distinguish competent from incompetent embryos. Defective decidualization—arising from progesterone resistance, chronic inflammation, or intrinsic stromal cell dysfunction—may therefore not only reduce baseline receptivity but also impair embryo quality control, potentially allowing the implantation of genetically abnormal embryos with downstream consequences for miscarriage risk [47,49] (Figure 1).

This selective mechanism has important clinical implications. It may explain why some women with recurrent implantation failure continue to fail despite transfer of euploid, morphologically high-grade embryos: the defect lies not in the embryo but in the endometrium’s capacity to respond to and sustain the implanting blastocyst. Conversely, it raises the possibility that an overly permissive endometrium—one that fails to reject compromised embryos—may contribute to biochemical pregnancy loss or first-trimester miscarriage. Therapeutic strategies aimed at optimizing decidualization, therefore, may improve not only implantation rates but also pregnancy continuation rates by restoring appropriate biosensor function.

## 6. Clinical Implications and Therapeutic Perspectives

Advances in reproductive immunology have catalyzed the development of diagnostic tools aimed at objectively assessing endometrial receptivity and identifying immune dysregulation. Transcriptomic profiling of the endometrium during the putative window of implantation—exemplified by the Endometrial Receptivity Array (ERA)—has been used to personalize embryo transfer timing based on the molecular clock of endometrial gene expression [9]. While this approach has demonstrated utility in a subgroup of patients with displaced implantation windows, particularly those with prior implantation failure, its broad applicability and cost-effectiveness remain subjects of ongoing clinical investigation [50]. Complementary approaches include immune profiling of endometrial biopsies for uNK cell quantification, Treg enumeration, and cytokine expression analysis, though standardization of sampling protocols and reference intervals remains an unmet need.

Progesterone remains the cornerstone of clinical management in both natural cycles and ART, supporting endometrial decidualization, immune tolerance, and uterine modulation [37]. Luteal phase progesterone supplementation is universally employed in IVF cycles, and progesterone resistance—a state of impaired endometrial responsiveness despite adequate circulating progesterone levels—is increasingly recognized as a contributor to implantation failure in conditions such as endometriosis and polycystic ovary syndrome. Immune-targeted therapies have been explored to address specific immunologic defects identified in women with recurrent implantation failure. Low-dose corticosteroids have been used to suppress uterine NK cell activity and dampen pro-inflammatory signaling, though randomized controlled trials have not consistently demonstrated benefit [51]. Intravenous immunoglobulin (IVIG) and intralipid infusions, postulated to modulate NK cell function through lipid-mediated mechanisms, have attracted considerable clinical interest, but their efficacy has not been established in well-powered prospective trials [52]. Tacrolimus is considered a possible treatment for women with elevated Th1/Th2 ratio [53]. However, one reason for aberrant T-helper cell functions is thought to be due to chronic endometritis. Thus, suppressing the T cell function using tacrolimus might not help in improving pregnancy rates until the primary defect is corrected, and antibiotics are proposed to be the first line of treatment [54]. Treatment with prednisolone, vitamin E, and intralipids was offered to women with recurrent implantation failure to suppress inflammation and oxidative stress [55,56]. The results were promising in some women with hyperactivated endometrium but there is a need for validation with large RCT studies. The absence of validated predictive biomarkers to select patients most likely to benefit from immune-targeted interventions represents a major barrier to their rational clinical application.

The endometrial microbiome has emerged as an additional modulator of implantation biology, challenging the long-held dogma of uterine sterility [57]. Metagenomic sequencing studies have demonstrated that the endometrium harbors a low-biomass but distinct microbial community, predominantly comprising Lactobacillus species in fertile women [58]. Lactobacillus-dominant endometrial microbiota are associated with higher IVF success rates, whereas a non-Lactobacillus-dominant profile—enriched with Gardnerella, Prevotella, or anaerobic species—correlates with reduced receptivity and increased implantation failure. The mechanisms underlying microbiome–endometrium interactions are incompletely understood but may involve modulation of local inflammatory tone, antimicrobial peptide production, and interaction with toll-like receptors on epithelial and immune cells. Probiotic interventions and vaginal microbiome optimization are being explored as therapeutic strategies, though evidence from randomized trials is currently limited. Future clinical approaches to recurrent implantation failure are likely to incorporate multi-omics profiling combining transcriptomic, immunologic, and microbiomic data to generate personalized treatment algorithms, moving the field toward precision reproductive medicine.

## 7. Discussion

Implantation is a complex, multifactorial process that demands the precise coordination of hormonal, cellular, molecular, and immunologic systems across a narrow temporal window. The body of evidence reviewed here collectively supports a model in which successful implantation depends on an active, bidirectional dialogue between the embryo and the endometrium, mediated by soluble factors, extracellular vesicles, and direct cell to cell interactions. A central conceptual advance is the recognition of the endometrium not as a passive scaffold, but as an active immunologic and biosensory organ capable of evaluating embryo quality and mounting differentiated responses that either support or reject the implanting conceptus [47].

Despite significant mechanistic progress, translation into clinical practice remains limited by several fundamental challenges. A persistent difficulty is distinguishing causative immune dysfunction from secondary changes that accompany implantation failure. Cross-sectional and retrospective study designs dominate the literature, making it difficult to establish causality rather than association. Furthermore, the heterogeneity of patient populations—encompassing women with diverse etiologies of implantation failure, ranging from endometriosis to unexplained infertility—limits the assessment of findings and the identification of universally applicable therapeutic strategies [50]. Variability in endometrial sampling protocols, timing relative to the menstrual cycle, laboratory methodologies, and the absence of standardized reference ranges for immune markers further hinder cross-study comparisons and clinical implementation.

Future research must address these limitations through prospective, well-powered clinical trials with pre-specified immune profiling protocols, standardized outcome definitions, and mechanistically informed patient stratification. Integrative multi-omics approaches—combining transcriptomics, proteomics, metabolomics, and microbiome analysis—hold the potential to identify composite biomarker signatures that predict receptivity and guide personalized therapeutic interventions (Table 1). Advances in single-cell RNA sequencing and transcriptomics are providing unprecedented resolution of the heterogeneity of the implantation microenvironment, revealing previously uncharacterized immune cell subsets and signaling interactions. The field is also beginning to incorporate artificial intelligence and machine learning tools for the integrative analysis of complex, multi-dimensional clinical and biological datasets, potentially enabling the development of predictive algorithms for implantation outcomes. Ultimately, the goal of precision reproductive medicine—delivering the right therapeutic intervention to the right patient at the right time—requires not only mechanistic insight but also the development of clinically validated, non-invasive or minimally invasive tools for endometrial and immune assessment.

## 8. Conclusions

Successful embryo implantation depends on a finely orchestrated dialogue between the blastocyst and the endometrium, encompassing immune regulation, molecular signaling, hormonal programming, and microbial modulation. The immunologic landscape of the receptive endometrium—dominated by uNK cells, Tregs, macrophages, and dendritic cells—is not static but dynamically regulated in response to both endogenous hormonal cues and exogenous embryo-derived signals. Intracellular signaling pathways including JAK/STAT, PI3K/AKT, NF-κB, and MAPK serve as molecular paths that integrate these diverse inputs to coordinate gene expression, cellular differentiation, and immune tolerance. The embryo–endometrium dialogue, mediated through hCG, cytokines, growth factors, and extracellular vesicles, is central to this process and represents the most dynamic and clinically underexplored dimension of implantation biology. Disruption of any component of this dialogue—whether through immune dysregulation, defective decidualization, impaired EV signaling, or altered microbiome composition—may result in implantation failure even when embryo quality is optimal. Recognizing this complexity is essential for moving beyond embryo-centric views of implantation failure toward a more holistic understanding of reproductive success. Translating these mechanistic insights into validated clinical diagnostic and therapeutic tools remains a key challenge and the defining opportunity of the next generation of reproductive medicine research.

## Figures and Tables

**Table 1:** Key Immunologic Components of Endometrial Receptivity.

Component	Function	Key Molecules	Clinical Relevance
uNK cells	Angiogenesis, trophoblast regulation	VEGF, PlGF, KIR	Implantation failure, preeclampsia
Tregs	Immune tolerance	FOXP3, IL-10, TGF-β	Recurrent implantation failure
Macrophages	Tissue remodeling	IL-10, growth factors	Decidualization defects
Dendritic cells	Immune regulation	Cytokines	Immune imbalance
Cytokines	Signaling regulation	LIF, TNF-α	Implantation outcomes
EVs	Intercellular communication	miRNAs, proteins	Emerging biomarkers

## Data Availability

The original contributions presented in this study are included in the article. Further inquiries can be directed to the corresponding author.

## Abbreviations

AKT	protein kinase B
ART	assisted reproductive technology
CSF-1	colony-stimulating factor-1
DC	dendritic cell
ERA	Endometrial Receptivity Array
EV	extracellular vesicle
FOXO	forkhead box O transcription factor
FOXP3	forkhead box P3
HB-EGF	heparin-binding epidermal growth factor-like growth factor
hCG	human chorionic gonadotropin
HLA	human leukocyte antigen
ICAM-1	intercellular adhesion molecule-1
IGF-1	insulin-like growth factor-1
IL-1β	interleukin 1 beta
IL-6	interleukin 6
IL-10	interleukin 10
IVIG	intravenous immunoglobulin
IVF	in vitro fertilization
JAK	Janus kinase
KIR	killer-cell immunoglobulin-like receptor
LHCGR	luteinizing hormone/chorionic gonadotropin receptor
LIF	leukemia inhibitory factor
MAPK	mitogen-activated protein kinase
miRNA	microRNA
MMP	matrix metalloproteinase
mRNA	messenger RNA
NF-κB	nuclear factor kappa B
NK	natural killer
PI3K	phosphoinositide 3-kinase
PlGF	placental growth factor
STAT3	signal transducer and activator of transcription 3
TGF-β	transforming growth factor beta
Th1	T helper type 1
Th2	T helper type 2
TNF-α	tumor necrosis factor alpha
Treg	regulatory T cell
uNK	uterine natural killer
VEGF	vascular endothelial growth factor
